# ALK-negative urachal inflammatory myofibroblastic tumor in an elderly female: A case report: Erratum

**DOI:** 10.1097/MD.0000000000014246

**Published:** 2019-01-18

In the article, “ALK-negative urachal inflammatory myofibroblastic tumor in an elderly female: A case report”,^[1]^ which appears in Volume 97, Issue 51 of *Medicine*, the corresponding author information should appear as:

Correspondence: Gongxian Wang, Departments of Urinary Surgery, The First Affiliated Hospital of Nanchang University, Nanchang, Jiangxi 330006, China (e-mail: wanggx-mr@126.com); Shuyan Xia, Tongji Hospital of Tongji Medical College, Huazhong University of Science and Technology, Wuhan, Hubei 430030, China (e-mail: xsy_0309@163.com).
